# Rutin-Loaded Silver Nanoparticles With Antithrombotic Function

**DOI:** 10.3389/fbioe.2020.598977

**Published:** 2020-11-25

**Authors:** Haitao Wu, Manlin Su, Hui Jin, Xinyu Li, Puyu Wang, Jingxiao Chen, Jinghua Chen

**Affiliations:** Key Laboratory of Carbohydrate Chemistry and Biotechnology, Ministry of Education, School of Pharmaceutical Sciences, Jiangnan University, Wuxi, China

**Keywords:** rutin, silver nanoparticles, antithrombosis, anticoagulant, sustained release

## Abstract

In this paper, we fabricated rutin-loaded silver nanoparticles (Rutin@AgNPs) as the nano-anticoagulant with antithrombotic function. The serum stability, anticoagulation activity, and bleeding risk of Rutin@AgNPs were evaluated. The results showed Rutin@AgNPs had good serum stability, hemocompatibility, and cytocompatibility. The anticoagulation activity of rutin was maintained, and its stability and aqueous solubility were improved. The Rutin@AgNPs could provide a sustained release to prolong the half-life of rutin. The results of the coagulation parameter assay and thrombus formation test in mice model showed that the activated partial thromboplastin time and prothrombin time were prolonged, and Rutin@AgNPs inhibited the thrombosis in the 48 h period. Moreover, the limited bleeding time indicated that the Rutin@AgNPs significantly minimized the hemorrhage risk of rutin. This Rutin@AgNPs is a potential anticoagulant for antithrombotic therapy.

## Introduction

Thromboembolism is one of the leading causes of cardiovascular diseases (Lippi et al., 2011; Gorog et al., 2017; Dakin, 2019). It leads to circulatory disorders, such as ischemic stroke, myocardial infarction, and pulmonary embolism, which are the major cause of morbidity and mortality (Kyrle et al., 2004). Previous researchers have found irregular coagulation and thrombus formation are the leading causes of thromboembolism (Mackman, 2008). To inhibit abnormal coagulation and thrombus formation, many researchers devote to anticoagulants development which can target blood clots related coagulation factors. Heparin and warfarin (vitamin K antagonist) are two mainstream anticoagulants that are used clinically. Heparin can activate antithrombin III and inhibit factor Xa. However, its half-life is short, and cannot be administered orally (Hirsh et al., 2001a). Moreover, this anticoagulant has a high risk of massive hemorrhage complications (Chen C. et al., 2016). To optimize the safety of heparin treatment, low molecular weight heparin (LMWH), which has a similar mechanism of action, has been developed. LMWH shows a better benefit-to-risk ratio but still cannot make up the complications (such as frequent thrombocytopenia and osteoporosis events) of heparin (Hirsh et al., 2001a, b). Warfarin is an anticoagulant that works on factors II, VII, IX, and X. It can effectively reduce the amount of prothrombin. However, apart from the hemorrhage complications, warfarin can cause a hypercoagulable state which results in thrombosis of the venules and capillaries, and leads to skin necrosis in the further stage (Ansell et al., 2008). The causes of these complications are anticoagulants over inhibit the coagulation. Therefore, researchers keenly aware an effective and safe antithrombotic target is needed. Recent studies have found the protein disulfide isomerase (PDI), which is expressed on the surface of platelets and endothelial cells, plays a critical role in thrombus formation (Jasuja et al., 2011, 2012; Furie and Flaumenhaft, 2014). Inhibition of PDI can affect both platelet aggregation and fibrin generation. Moreover, it is a safe target for the inhibition of thrombus formation (Flaumenhaft, 2013; Kennedy et al., 2013). Quercetin-3-rutinoside (rutin) is a kind of flavonoid glycoside, which is abundant in buckwheat, tea, fruits, and berries (Gullon et al., 2017). Studies have found that rutin is a potential thromboprophylaxis agent, and can be used as an effective inhibitor of PDI both *in vitro* and *in vivo* (Jasuja et al., 2012; Flaumenhaft, 2013; Choi et al., 2015; Lin et al., 2015). As a prescribed traditional medicine and an antioxidant, rutin has been proved to be toxicologically safe and well-tolerated (Sharma et al., 2013). However, the poor bioavailability of rutin, which associates with stability and aqueous solubility, limited its clinical applications (Gullon et al., 2017). Thence, the key, which to develop rutin as an effective anticoagulant, is to improve the bioavailability of the rutin in blood.

**FIGURE 1:**
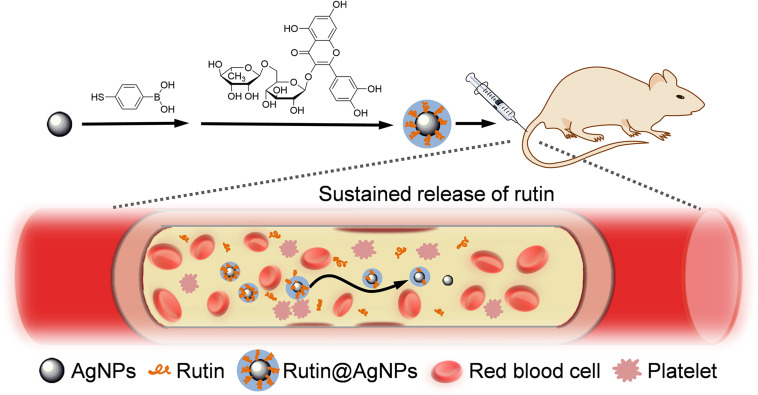
Construction of Rutin@AgNPs for long-term rutin release and antithrombotic therapy.

Nanomedicine is a popular research topic recently. Engineering nanoparticles (NPs) have been used as nanocarriers to load and to deliver anticoagulant toward the target directly (Tian et al., 2014; Su et al., 2020). They reduce the dose of the anticoagulant while improving the antithrombotic efficacy, and can decrease the hemorrhage complications (Ilinskaya and Dobrovolskaia, 2013a, b). The nanocarrier also can be used to improve the stability and aqueous solubility of the therapeutic agents (Kargl and Kleinschek, 2020). Silver nanoparticles (AgNPs) is widely used as potent antibacterial agents, as it has good biocompatibility. The size, shape, and architecture of AgNPs can be regulated, and surface modification of AgNPs can be used for drug loading in simple and convenient methods. Therefore, this study combined AgNPs and rutin to improve the stability and aqueous solubility of rutin. Here, the phenylboronic acid (PBA) group was chemically introduced onto the surface of AgNPs at first. Then, rutin was loaded on AgNPs to form the Rutin@AgNPs through a dynamic boronate ester bond (Chen J.X. et al., 2016), as shown in Figure 1. We expected the Rutin@AgNPs could increase the solubility and stability of rutin, and prolong the half-life of rutin through a sustained release strategy.

## Materials and Methods

### Materials

Rutin and 4-mercaptophenylboronic acid (MPBA) were obtained from Aladdin Reagent Co. (China). Silver nitrate, sodium borohydride (NaBH_4_), NH_4_HCO_3_, and dimethyl sulfoxide (DMSO) were purchased from Sinopharm Chemical Reagent Co., Ltd. (China). Heparin sodium was provided by Sangon Biotech (Shanghai) Co., Ltd. Polyvinylpyrrolidone (PVP, Mw: 10 kDa), carrageenan, and urethane were obtained from Sigma-Aldrich Co. (United States). Endothelial cell basal medium (ECM), fetal bovine serum (FBS), and 3-(4,5-Dimethylthiazol-2-yl)-2,5-diphenyltetrazoliumbromide (MTT) were purchased from Thermo Fisher Scientific Co. (United States).

### Preparation of Rutin@AgNPs

AgNPs were prepared through a reduction method under the stabilization of PVP (Tejamaya et al., 2012). Briefly, 10 mL of AgNO_3_ solution (1.02 mg/mL) was added into 60 mL of PVP solution (1.5 mg/mL) under stirring. Then, 10 mL of NaBH_4_ solution (0.6 mg/mL) was added dropwise into the solution. A light-brown AgNPs solution was obtained after vigorous stirring for 15 min. After that, 0.4 mL of MPBA (2 mg/mL) in DMSO was added into the AgNPs solution. The solution turned brown, and PBA modified AgNPs (PBA@AgNPs) was obtained after stirring for 2 h. Subsequently, the pH of the solution was adjusted to about 8. Then, 2 mL of rutin (10 mg/mL) in DMSO was added to this solution. After stirring for 4 h, the solution was put into a dialysis tube (molecular weight cut-off, MWCO: 14 kDa) and subjected to dialysis against NH_4_HCO_3_ solution (pH 8.0) for 2 days to remove the organic solvent and other impurities, followed by lyophilization to collect Rutin@AgNPs.

### Characterizations of Rutin@AgNPs

The composition of Rutin@AgNPs was identified by the Fourier transform infrared spectroscopy (FTIR) via a TENSOR II spectrometer (Bruker, Germany). The loading amount of rutin on AgNPs was measured by using thermogravimetric analysis (TGA) on a TGA/1100SF instrument (Mettler-Toledo, Switzerland). The flow rate of N_2_ was 50 mL/min, the heating rate was 10^*o*^C/min. The freeze-dried AgNPs, PBA@AgNPs, and Rutin@AgNPs were resolved in phosphate buffer saline (PBS, pH 7.4) at the concentration of 1 mg/mL. The ultraviolet-visible light (UV-vis) absorption of rutin and these NPs was measured on a UV 2550 UV-vis spectrophotometer (Shimadzu, Japan) at the wavelength ranges from 200 to 700 nm.

The size distributions of three types of NPs were measured by using a dynamic light scattering technique on a Zetasizer Nano ZS apparatus (Malvern, United Kingdom). The morphology of three types of NPs was observed by using transmission electron microscopy (TEM) on a JEM-2100 instrument (JEOL, Japan) with an accelerating voltage of 80 kV. The particle size in the TEM images was gained by using Image J software.

### *In vitro* Drug Release Assay

Briefly, 10 mL Rutin@AgNPs solution (1 mg/mL) was put into different dialysis tubes (MWCO: 3,500 Da). Next, the dialysis tubes were immersed into 20 mL of PBS (pH 7.4), which excluded/included glucose (5.5 mmol/L, 20 mmol/L). Then, the dialysis tubes were placed in the 37^*o*^C shaking bath to allow the release of rutin. At the assigned time interval, the samples were withdrawn, and the PBS was refreshed. The amount of released rutin in the medium was determined by UV-vis spectrophotometer at 262 nm. The cumulative release ratio of rutin was calculated according to the equation below. Three independent trials were carried out, and their average results were used.

$$Cumulativerelease\left(\%\right)=\frac{20}{2.83}\times \sum_{i=1}^{n}C_{i}\times 100\%$$

where C_*i*_ represents the rutin concentration of sample i, n is the total number of samples.

### Stability and Hemolysis Test

The serum stability of the Rutin@AgNPs was determined through the UV-vis spectrum and particle size distribution, where the Rutin@AgNPs solutions contain 10% FBS (v/v) were placed in the dark environment for 3 days before the test.

The hemocompatibility of the Rutin@AgNPs was evaluated through the hemolytic rate. The fresh blood sample (10 mL) was placed in K_2_-EDTA-coated Vacutainer tubes firstly. Next, the red blood cells (RBCs) were gained through centrifugation (2,500 × g for 10 min) and washed with PBS (pH 7.4) three times. Then, the RBCs were suspended into 7 × 10^9^ cells/mL with PBS and were distributed into six sample groups (2 mL each). Four groups of the samples were added Rutin@AgNPs in different concentrations (10, 50, 100, and 1,000 mg/L). The PBS and the PBS containing 20% Triton X-100 were added into the negative control group and positive control group, respectively. All the sample groups were incubated in a water bath (37^*o*^C) on a shaker for 1 h. After centrifugation (2,500 × g for 10 min), the supernatant’s UV-vis absorption of the test sample group (A), the negative control group (A_0_), and the positive control group (A_100_) were measured at 545 nm. These three measurement results were used to calculate the hemolytic rate, as shown in the equation below. Note here, three independent trials were carried out, and their average results were used.

$$\text{Hemolytic}\mathrm{rate}\left(\%\right)=\frac{A-A_{0}}{A_{100}-A_{0}}\times 100\%$$

### *In vitro* Cytotoxicity Assay

The human umbilical vein endothelial cells (HUVECs) were cultured in ECM (containing 10% FBS) at 37^*o*^C in a humidified condition containing 5% CO_2_. The cytotoxicity of rutin, AgNPs, and Rutin@AgNPs were tested by MTT assay using HUVECs as the model cell. Briefly, the HUVECs were seeded on a 96 well-plate (5,000 cell/well) and cultured overnight. Then, rutin solution with different concentrations (1.25, 6.25, 12.5, 25, 75, and 125 mg/L, containing 5% DMSO), AgNPs and Rutin@AgNPs (10, 50, 100, 200, 600, and 1,000 mg/L) were added, respectively. After 48 h, the cultural mediums were replaced by 100 μL MTT solution (0.5 mg/mL), and add in 100 μL DMSO after another 4 h. The optical density (OD) at 570 nm was measured by using a Multiskan MK3 microplate reader (Thermo Fisher Scientific United States). The cell viability rate was calculated by using the equation below. The data was obtained from the average value of three independent trials.

$$\text{Cell}\text{viability}\left(\%\right)=\left(\frac{OD_{sample}}{OD_{control}}\times 100\%\right)$$

where the OD_sample_ is the OD value of the presence of samples, the OD_control_ is the OD value of control well with absence samples.

### Animals

ICR mice (5-weeks old, 28–32 g) were obtained from SLAC Laboratory Animal Co., Ltd., Shanghai, China). All animal care and experiments were conducted following the guidelines of the Institutional Animal Care and Use Committee of Jiangnan University.

### *In vivo* Coagulation Parameter Assay

Briefly, 36 ICR mice were randomly divided into six groups (*n* = 6). Group 1–6 were intravenously injected through mice tail with saline (control group), AgNPs (600 μg/kg), Rutin@AgNPs (300 μg/kg), Rutin@AgNPs (600 μg/kg), rutin (200 μg/kg), and heparin (500 μg/kg), respectively. Administration for 1 h, the blood was drawn from the mice and added a 3.8% citrate solution (1:9 citrate/blood, v/v) to avoid coagulation. Finally, the activated partial thromboplastin time (aPTT) and prothrombin time (PT) were measured by a coagulometer (Sysmex CA-8,000, Japan) (Tian et al., 2014).

### Carrageenan-Induced Tail Thrombosis Assay

Thirty mice were randomly divided into five groups (*n* = 6). Group 1–5 were intravenously injected through mice tail with saline, AgNPs (600 μg/kg), Rutin@AgNPs (600 μg/kg), rutin (200 μg/kg), and heparin (500 μg/kg), respectively. After 1 h of the intravenous administration, 1% carrageenan (50 mg/kg) was intraperitoneally injected into mice to induce the tail thrombosis model (Hagimori et al., 2009). The length of the tail thrombus was measured twice, after 24 and after 48 h.

### Mouse Tail Bleeding Test

The mouse tail bleeding test was used to evaluate the thrombus formation (Dejana et al., 1982). Thirty mice were randomly divided into 5 groups (*n* = 6). Group 1 was set as a control group, which was injected with saline. Groups 2, 3, 4, and 5 were intravenously injected through mice tail with AgNPs (600 μg/kg), Rutin@AgNPs (600 μg/kg), rutin (200 μg/kg), and heparin (500 μg/kg), respectively. After administration for 1 h, urethane (20%, w/v, 0.1 mL/10 g) was intraperitoneally injected into each mouse for anesthesia. The mouse tail was cut 1 cm from the rare, then we started the timing. The bleed out blood was removed every 20 s until coagulation. The timing stopped and recorded it as the bleeding time.

### Statistical Analysis

All data were presented with mean ± SD. One-way ANOVA *post hoc* tests were performed by using SPSS 23.0 software, and the significance level was set at 0.05. One (^∗^) and two (^∗∗^) represent *p* < 0.05 and *p* < 0.01, respectively.

## Results and Discussion

### Composition of Rutin@AgNPs

**FIGURE 2:**
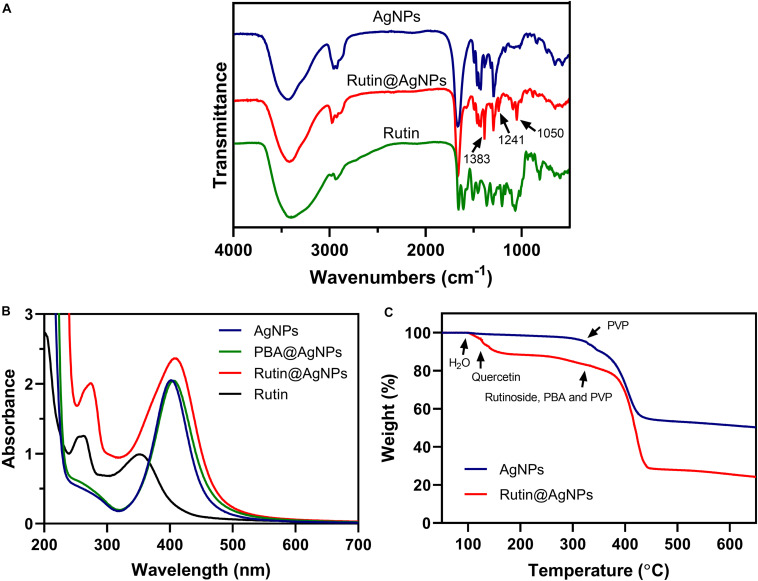
**(A)** FTIR spectra of AgNPs, Rutin@AgNPs, and rutin; **(B)** UV-vis spectra of AgNPs, PBA@AgNPs, Rutin@AgNPs, and rutin; **(C)** TGA curves of AgNPs and Rutin@AgNPs.

Chemical modification is an important strategy to functionalize NPs. It varies the composition and physicochemical properties of NPs and enriches their applications. Due to the poor thermostability of the rutin, we tried to find a gentle manner to load rutin on the AgNPs. We noticed that rutin is composed of quercetin (including catechol structure) and glycoside, which possess 1,2-diol functional group. This inspired us to choose boronate ester (a dynamic covalent bond) to load rutin on the AgNPs. Boronate ester bond can form between phenylboronic acid and 1,2-diol in a weak alkaline environment (pH 8.0) at room temperature (25^*o*^C). Since the thermal decomposition temperature of rutin is higher than 70^*o*^C (Chaaban et al., 2017), this reaction is gentle and straightforward for rutin loading. The composition of Rutin@AgNPs was first confirmed by FTIR and UV-vis spectra. As shown in Figure 2A, the peaks appeared at 3,400, 2,935, and 1,650 cm^–1^ in the FTIR spectrum of rutin belonged to −OH, CH_2_, and C = O stretching vibrations, respectively. These peaks also appeared in the FTIR spectrum of AgNPs, but they belonged to the PVP that coated on the outer layer of AgNPs (Zhang et al., 2011). The characteristic peak at 1,450 cm^–1^ exhibited in spectra of both AgNPs and Rutin@AgNPs belonged to the C-N stretching vibration of PVP, indicated that the PVP was also coating on the outer surface of Rutin@AgNPs. For the FTIR spectra of Rutin@AgNPs and Rutin, the peaks appeared at 1,383 and 1,050 cm^–1^ belonged to the C-OH stretching vibrations of quercetin structure and glycoside structure, respectively (Zahoor et al., 2018). This result not only suggested rutin was successfully loaded on the AgNPs, but showed the rutin structure remained complete as well. What’s more, a newly generated peak at 1,241 cm^–1^ only appeared in the FTIR spectrum of Rutin@AgNPs. This peak was ascribed to the C-O stretching vibration and served as a solid proof of the boronate ester formation between PBA and catechol in rutin (Thompson et al., 2017). As shown in Figure 2B, the maximum absorption of rutin in the UV-vis spectrum was at 262 nm. Comparing with the spectra of three NPs, this absorption appeared in the spectrum of Rutin@AgNPs and red-shifted to 273 nm. This was due to the loaded rutin gather on the surface of the AgNPs that would be influenced by the plasmonic resonance of AgNPs. These results also proved that rutin was loaded successfully.

The rutin loading amount was evaluated through the percentage of weight loss, as shown in Figure 2C. Comparing the thermal decomposition process of the AgNPs and Rutin@AgNPs, the Rutin@AgNPs showed a stepwise thermal decomposition, but AgNPs only contained one stage. In the AgNPs curve, the stage began at about 320^*o*^C was attributed to the thermal decomposition of coated PVP. In the Rutin@AgNPs curve, the decomposition stages belonged to water, quercetin, and rutinoside of the rutin, the PBA, and the PVP (da Costa et al., 2002; Wang et al., 2015). The first stage appeared at 100–123^*o*^C belonged to the absorbed water (about 3.4% in weight). The second stage exhibited at 123–320^*o*^C ascribed to the decomposition of quercetin (about 13.2% in weight). The stage that appeared beyond 320^*o*^C belonged to the decomposition of rutinoside, the PBA, and the PVP. As the weight loss of rutinoside was mixed with that of the PBA and the PVP, the loading amount of the rutin was determined through the weight loss of quercetin only. Therefore, the loading amount of rutin on Rutin@AgNPs was calculated as 28.3%, by analyzing the quercetin content in rutin through the molecular weight.

### Morphology of Rutin@AgNPs

**FIGURE 3:**
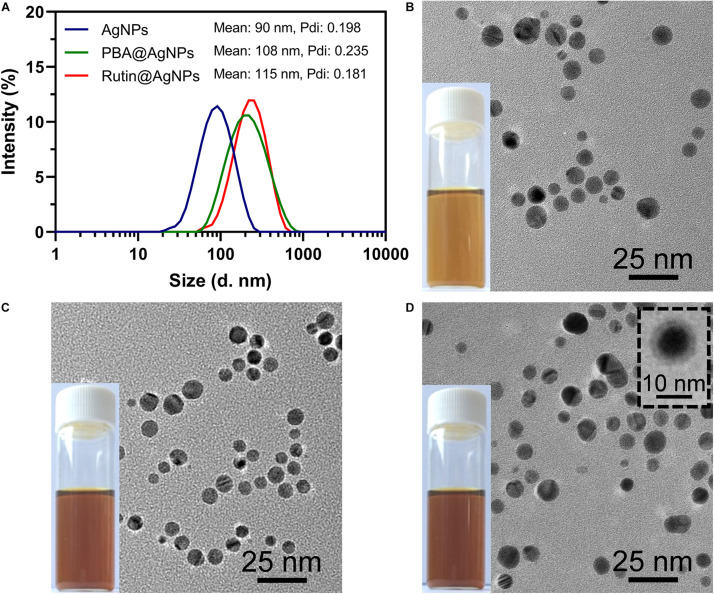
**(A)** Size distributions of three types of NPs; TEM images and photographs of **(B)** AgNPs, **(C)** PBA@AgNPs, and **(D)** Rutin@AgNPs. The insert image showed Rutin@AgNPs were negatively stained with phosphotungstic acid.

The morphology of Rutin@AgNPs was investigated by using DLS and TEM (Figure 3). The size distributions and the average hydrodynamic diameters of AgNPs, PBA@AgNPs, and Rutin@AgNPs were shown in Figure 3A. Comparing the size distributions of three types of NPs, the average diameters of PBA@AgNPs and Rutin@AgNPs were larger than that of AgNPs, but their distribution ranges remained visually constant. The mean hydrodynamic diameters of AgNPs, PBA@AgNPs, and Rutin@AgNPs were 90, 108, and 115 nm, respectively. Their polydispersity index values were at 0.198, 0.235, and 0.181, respectively. These values were all below 0.3, which indicated that three NPs were homogenous, and Rutin@AgNPs was acceptable for rutin delivery (Danaei et al., 2018). Also, the values inferred that the enlargement of AgNPs was along with the PBA and rutin successively decoration. The morphological properties of these NPs were further investigated by TEM. All three NPs appeared to have a homogenously spherical shape and a well-dispersed feature, as shown in Figures 3B–D. The average size of AgNPs and Rutin@AgNPs, which evaluated by Image J, were 12.2 ± 1.3 nm and 14.5 ± 1.8 nm, respectively. They exhibited a similar rising trend of average diameter in the results of DLS. Note here, the size in TEM was relatively smaller than the one in DLS. This was attributed to the PVP outer layer of NPs, which were spread in the aqueous medium but shrunken in a dry state. The Rutin@AgNPs was further negatively stained with phosphotungstic acid. A magnified image of it is inserted in Figure 3D. In this image, the coated PVP and loaded rutin at the outer hydrophilic layer can be identified visually. The results above proved the prepared NPs achieved successfully.

### *In vitro* Rutin Release of Rutin@AgNPs

**FIGURE 4:**
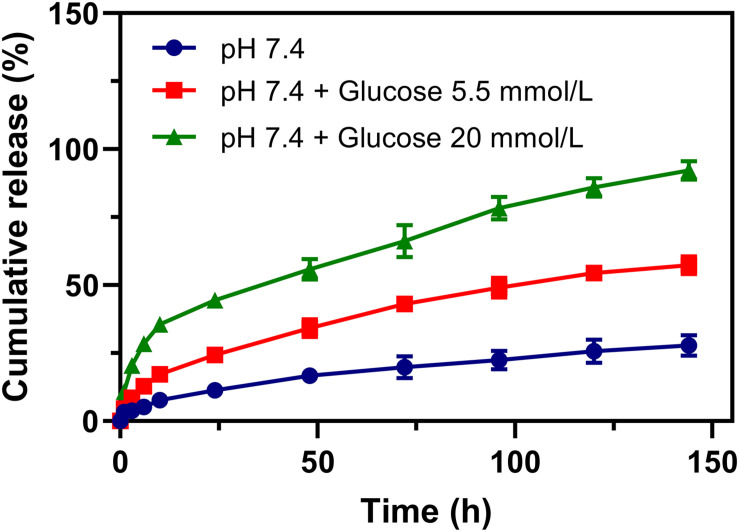
Rutin release profiles of Rutin@AgNPs in PBS (pH 7.4) excluded or included glucose (5.5 and 20 mmol/L).

The release of rutin in the physiological environment is the precondition of Rutin@AgNPs to be an anticoagulant. The rutin release was investigated in different simulated physiological environments, which were the PBS and the PBS containing glucose (5.5 and 20 mmol/L). In Figure 4, the cumulative release rate of rutin in 6 days was 27.9 and 57.3%, for Rutin@AgNPs in the PBS and the PBS containing glucose (5.5 mmol/L), respectively. The release rate of rutin accelerated in the PBS containing glucose. As the boronate ester bond was glucose sensitive (Roy and Sumerlin, 2012; Zhao et al., 2017), it would dynamically occur glucose exchange in a hyperglycemia environment. To verify this speculation, the rutin release was further carried out in a PBS containing glucose (20 mmol/L). The release rate of rutin obviously increased, and the cumulative release amount of rutin reached 92.1% in 6 days. Since venous thromboembolism is one of the common complications of diabetes (Piazza et al., 2012; Bell et al., 2016), the Rutin@AgNPs, which had a glucose-sensitive rutin release feature, was potentially applied in diabetes-related venous thromboembolism therapy. More importantly, the rutin release curves in the simulated physiological environments were smooth and continuous, close to the zero-order sustained release behavior. This finding indicated that the Rutin@AgNPs provided a steady-state of rutin concentration in blood circulation, which might overcome the short half-life barrier of rutin.

### Serum Stability and Biocompatibility of Rutin@AgNPs

**FIGURE 5:**
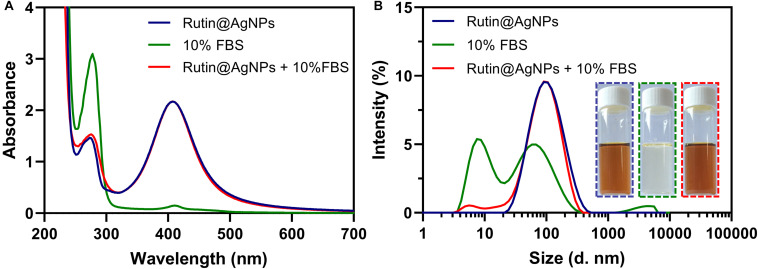
**(A)** UV-vis spectra, **(B)** size distributions, and photographs of Rutin@AgNPs cultivated in the solution containing 10% FBS for 3 days.

The stability of NPs in blood circulation is one of the fundamental conditions for an antithrombotic trial. The serum stability of Rutin@AgNPs was investigated through UV-vis spectra and size distributions. In Figure 5A, two UV-vis spectra, which related to the Rutin@AgNPs, were overlapped at above 320 nm, but showed different absorption peaks below 320 nm. These two absorption peaks were at 273 and 277 nm, for Rutin@AgNPs and Rutin@AgNPs cultivated with FBS (Rutin@AgNPs + FBS), respectively. The FBS curve showed a characteristic absorption peak at 277 nm, which appeared at the same wavelength of the Rutin@AgNPs + FBS curve. This implied that the peak of the Rutin@AgNPs + FBS curve at 277 nm appeared as the addition of the FBS solution. Since the curves related to the Rutin@AgNPs overlapped above 320 nm, the results inferred that the size change of the NP was limited. Comparing the size distributions of Rutin@AgNPs and Rutin@AgNPs + FBS, two curves illustrated a good match between 30 and 200 nm, as shown in Figure 5B. The peak appeared at about 2–10 nm of Rutin@AgNPs + FBS could attribute to the particles in the FBS. These findings indicated that Rutin@AgNPs was stable and could resist serum binding and serum-induced aggregation in the solution containing FBS, which revealed good serum stability of Rutin@AgNPs.

**FIGURE 6:**
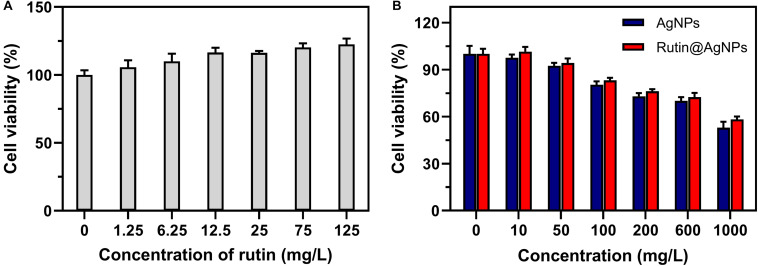
Cell viability of HUVECs cultured with **(A)** rutin, **(B)** AgNPs, and Rutin@AgNPs at different concentrations.

Hemolysis and cytocompatibility are two important indices for the NP to use in the blood circulation. So, experiments were carried out *in vitro* to evaluate the hemocompatibility and cytocompatibility of Rutin@AgNPs. The hemolytic rates, as listed in Table 1, showed that the hemolysis raised along with the increasing Rutin@AgNPs concentration. Even though the hemolysis showed a rising trend, the hemolytic rate only exceeded 5%, when the Rutin@AgNPs concentration above 1,000 mg/L. This implied that the Rutin@AgNPs had a satisfactory concentration range to meet the requirement of hemocompatibility (International Organization for Standardization, 2017). The cytocompatibility was evaluated by the MTT assay. In Figure 6A, the growth of HUVECs increased with the increasing concentration of rutin. This revealed that rutin is a kind of flavonoid with good cytocompatibility (Gullon et al., 2017). The cell viability rate of HUVECs gradually reduced while the increasing concentration of AgNPs and Rutin@AgNPs, as shown in Figure 6B. But the cell viability rates were above 80% when the NPs concentration reached 100 mg/L. Therefore, both NPs satisfied the safety requirement of biomedical applications (International Organization for Standardization, 2009). These also implied that both AgNPs and Rutin@AgNPs had reliable cytocompatibility. The concentration of Rutin@AgNPs below 100 mg/L was selected to use in further experiments.

**TABLE 1:** Hemolysis of human erythrocytes at various concentrations of Rutin@AgNPs.

C (mg/L)	10	50	100	1,000
Hemolysis (%)	0 ± 0	0.1 ± 0.01	0.4 ± 0.05	4.8 ± 0.12

### Effect of Rutin@AgNPs on Coagulation *in vivo*

**FIGURE 7:**
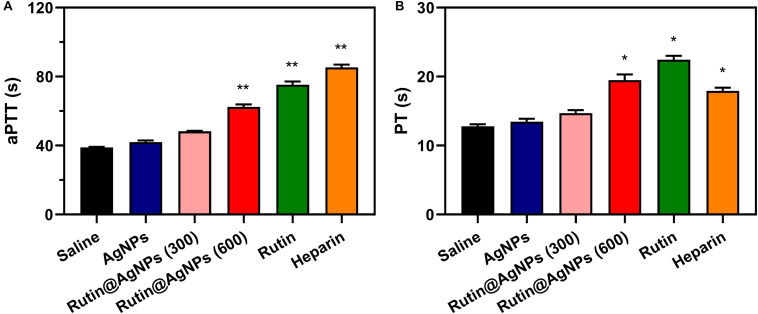
Effect of Rutin@AgNPs on **(A)** aPTT and **(B)** PT *in vivo*. Each value represents the mean ± SD (*n* = 6 for each test). *p < 0.05, **p < 0.01 as compared to values in the saline control group by one-way ANOVA *post hoc* tests.

Coagulation time is an intuitive assay for the anticoagulant activity evaluation. In this study, the *in vivo* aPTT and PT were tested after 1 h intravenous injection of Rutin@AgNPs in a mouse model. The aPTT and PT in the saline control group were 38.9 and 12.8 s, respectively, as shown in Figure 7. Comparing the coagulation time of two groups, the saline and the AgNPs, limited differences in aPTT or PT results were found. This implied that the AgNPs exhibited low anticoagulant activity. The aPTT and PT results of the rutin group were 75.3 s (*p* < 0.01) and 22.45 (*p* < 0.05), respectively. In comparison with the saline group, results of the rutin group increased by 1.9- and 1.7- fold, for aPTT and PT results, respectively. These findings indicated that the rutin had good anticoagulant activity and could prevent coagulation in both intrinsic and extrinsic clotting pathways. The aPTT and PT results of two rutin@AgNPs groups were longer than the saline group and AgNPs group. This implied the rutin@AgNPs was a promising anticoagulant, as it maintained the anticoagulation activity of rutin. Comparing two Rutin@AgNPs groups, the coagulation time raised when the dose increased. This inferred the coagulation time of the Rutin@AgNPs was dose-dependent. The results of the Rutin@AgNPs (600 μg/kg) showed a significant increase in the coagulation time (*p* < 0.01). This statistical analysis results also indicated that the coagulation time of the Rutin@AgNPs (600 μg/kg) group was competitive to the rutin group and heparin group. So, the Rutin@AgNPs (600 μg/kg) was selected to use in the further *in vivo* experiments.

### Antithrombus Formation

**FIGURE 8:**
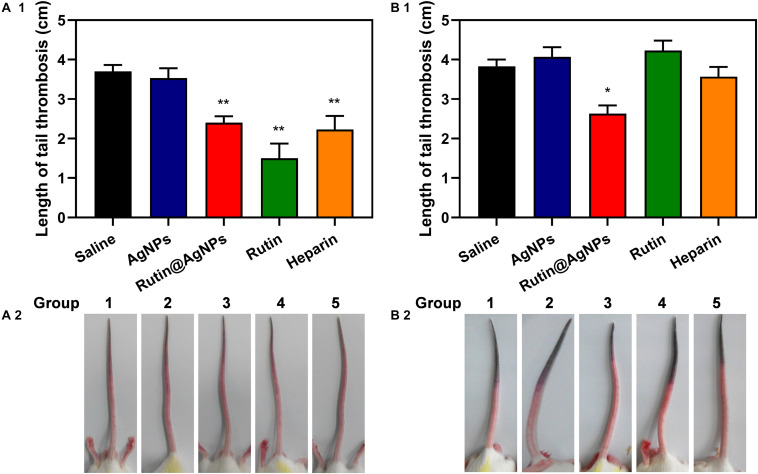
Effect of Rutin@AgNPs on carrageenan-induced tail thrombosis in mice for **(A1,A2)** 24 and **(B1,B2)** 48 h. Each value is shown as the mean ± SD (*n* = 6). *p < 0.05, **p < 0.01 as compared to values in the saline control by one-way ANOVA *post hoc* tests.

The carrageenan-induced venous thrombosis model in mouse was used in this study. This model was built by activating the Hageman (XII) factor, which was the cause of the platelet activation and aggregation, through intraperitoneal injection of carrageenan. The thrombus length that appeared on the mouse tails was used for evaluating the antithrombotic formation results. The results of 24 h and 48 h after injection were shown in Figure 8. For the results of 24 h after injection (Figure 8A), the thrombus length of the saline group and AgNPs group were similar, which were 3.7 and 3.5 cm, respectively. On the contrary, the thrombus length of the other three groups, Rutin@AgNPs, rutin, and heparin, were shorter, which were 2.4, 1.5, and 2.2 cm (*p* < 0.01), respectively. By comparing these results with the saline group and the AgNPs group, the thrombus formation of these three groups was inhibited. Here, the rutin group was the most effective. This was because rutin effectively targets the PDI and inhibits its activity, thus the platelet accumulation and fibrin generation can be blocked. Moreover, rutin is a kind of flavonoid, which contains antioxidation and anti-inflammatory activities. These activities reduced the inflammation which was caused by carrageenan, and decreased thrombus formation. For the results of after 48 h injections, the thrombus lengths of all the groups were increased, except the Rutin@AgNPs group (thrombus length = 2.6 cm, *p* < 0.05). The rutin group and heparin group, which showed promising results at the first 24 h, had their thrombus lengths increased greatly in the second 24 h. We inferred that the heparin and rutin had relatively shorter half-life limitation, and could degrade or metabolize *in vivo*, so their activities were lost over time. The Rutin@AgNPs inhibited thrombus formation for relatively longer in time, as it could sustainably release the rutin. This made up the fast degradation of the heparin and rutin. Note that, the rutin dose in the Rutin@AgNPs group was 169.8 μg/kg, which was lower than the dose in the rutin group. These findings indicated that Rutin@AgNPs can make up the half-life limitation of rutin, also increase its overall activities. This further implied that the Rutin@AgNPs had the potential to be used as a long-term antithrombotic agent.

### Bleeding Time Evaluation

**FIGURE 9:**
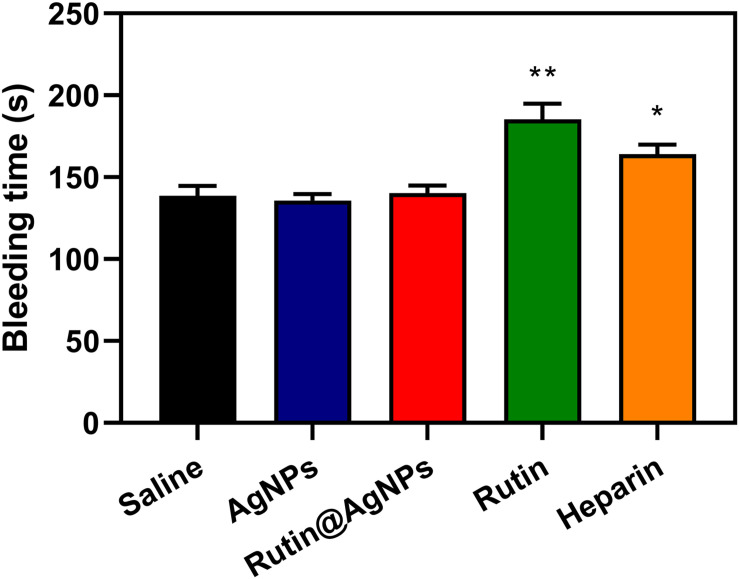
Effect of Rutin@AgNPs on bleeding time in mice. Each value is expressed as mean SD (n 6). *p 0.05, **p 0.01 as compared to values in the saline control by one-way ANOVA post hoc tests.

Bleeding is one of the severe complications of the anticoagulants in clinical trials, and the bleed time is commonly used as a safety evaluation parameter. A mouse tail-transection model was used to determine the bleeding time of Rutin@AgNPs in this study. The bleeding times of five injection sample groups (after 1 h) were illustrated in Figure 9. The saline and AgNPs groups’ results were similar at about 138.7 s. This was because the AgNPs had limited influence on the coagulation function (see section “Effect of Rutin@AgNPs on Coagulation *in vivo*”). The bleeding times of the rutin group and heparin group were 185.3 s (*p* < 0.01) and 164.2 s (*p* < 0.05), respectively, which were longer than the saline group. This implied that the rutin and heparin caused bleeding complications of anticoagulation, which lead to coagulation inhibitor disorder potentially. The bleeding time of the Rutin@AgNPs group was 140.3 s, which was slightly longer than that of the saline group. But the increment was not statistically significant (*p* > 0.5). Therefore, the Rutin@AgNPs had a limited effect on the normal coagulant function when it was used under a specific dose. From the above, we can infer that the sustained release property of the Rutin@AgNPs controlled the dose of the rutin in the blood. So, the long-term antithrombotic activity can be achieved, and the bleeding risk can be minimized.

## Conclusion

In this paper, we proposed a new NP, Rutin@AgNPs, which loaded rutin (calculated as 28.3%) on the surface of AgNPs, to control rutin release over time. This NP showed good serum stability, satisfiable hemocompatibility, and cytocompatibility, when concentration below 100 mg/L. The rutin was loaded through a dynamic boronate ester bond, which was glucose-sensitive. This assisted a sustained rutin release in the physiological environment. *In vivo* evaluations showed that Rutin@AgNPs maintained the anticoagulant function of rutin, prolonged the coagulation time in both intrinsic and extrinsic pathways. Comparing with rutin, Rutin@AgNPs not only inhibited the mouse tail thrombosis with a longer time, and solved the short half-life problem of rutin, but minimized bleeding risk as well. These were valid proof that the Rutin@AgNPs had satisfiable biocompatibility, and had a promising future in long-term antithrombotic therapy.

## Data Availability Statement

The original contributions presented in the study are included in the article/supplementary material, further inquiries can be directed to the corresponding author/s.

## Ethics Statement

The animal study was reviewed and approved by the Institutional Animal Care and Use Committee of Jiangnan University.

## Author Contributions

HW developed the main study. MS and HJ helped to complete the animal experiments. XL and PW took part in the preparation and analysis. JXC and JHC drafted the manuscript and developed the study design. All authors have given final approval for this manuscript to be published.

## Conflict of Interest

The authors declare that the research was conducted in the absence of any commercial or financial relationships that could be construed as a potential conflict of interest.

## References

[B1] Ansell J. Hirsh J. Hylek E. Jacobson A. Crowther M. Palareti G. (2008). Pharmacology and management of the vitamin K antagonists. *Chest* 133 160S–198S. 10.1378/chest.08-0670 18574265

[B2] Bell E. J. Folsom A. R. Lutsey P. L. Selvin E. Zakai N. A. Cushman M. (2016). Diabetes mellitus and venous thromboembolism: a systematic review and meta-analysis. *Diabetes Res. Clin. Pract.* 111 10–18. 10.1016/j.diabres.2015.10.019 26612139 PMC4752919

[B3] Chaaban H. Ioannou I. Chebil L. Slimane M. Gerardin C. Paris C. (2017). Effect of heat processing on thermal stability and antioxidant activity of six flavonoids. *J. Food Process. Preserv.* 41:e13203 10.1111/jfpp.13203

[B4] Chen C. Li S. Liu K. Ma G. Yan X. (2016). Co-assembly of heparin and polypeptide hybrid nanoparticles for biomimetic delivery and anti-thrombus therapy. *Small* 12 4719–4725. 10.1002/smll.201600328 27043722

[B5] Chen J.-X. Shi Y. Zhang Y.-R. Teng L.-P. Chen J.-H. (2016). One-pot construction of boronate ester based pH-responsive micelle for combined cancer therapy. *Colloids Surf. B Biointerfaces* 143 285–292. 10.1016/j.colsurfb.2016.03.053 27022868

[B6] Choi J.-H. Kim D.-W. Park S.-E. Lee H.-J. Kim K.-M. Kim K.-J. (2015). Anti-thrombotic effect of rutin isolated from Dendropanax morbifera Leveille. *J. Biosci. Bioeng.* 120 181–186. 10.1016/j.jbiosc.2014.12.012 25777266

[B7] da Costa E. M. Barbosa J. M. do Nascimento T. G. Macedo R. O. (2002). Thermal characterization of the quercetin and rutin flavonoids. *Thermochim. Acta* 392 79–84. 10.1016/s0040-6031(02)00087-4

[B8] Dakin S. G. (2019). Resolving deep vein thrombosis. *Sci. Transl. Med.* 11:eaay7696 10.1126/scitranslmed.aay7696

[B9] Danaei M. Dehghankhold M. Ataei S. Davarani F. H. Javanmard R. Dokhani A. (2018). Impact of particle size and polydispersity index on the clinical applications of lipidic nanocarrier systems. *Pharmaceutics* 10:57. 10.3390/pharmaceutics10020057 29783687 PMC6027495

[B10] Dejana E. Villa S. de Gaetano G. (1982). Bleeding time in rats: a comparison of different experimental conditions. *Thromb. Haemost* 48 108–111. 10.1055/s-0038-1657230 6753230

[B11] Flaumenhaft R. (2013). Protein disulfide isomerase as an antithrombotic target. *Trends Cardiovasc. Med.* 23 264–268. 10.1016/j.tcm.2013.03.001 23541171 PMC3701031

[B12] Furie B. Flaumenhaft R. (2014). Thiol Isomerases in Thrombus Formation. *Circ. Res.* 114 1162–1173. 10.1161/circresaha.114.301808 24677236 PMC4067134

[B13] Gorog D. A. Fayad Z. A. Fuster V. (2017). Arterial thrombus stability: does it matter and can we detect it? *J. Am. Coll. Cardiol.* 70 2036–2047. 10.1016/j.jacc.2017.08.065 29025561

[B14] Gullon B. Lu-Chau T. A. Moreira M. T. Lema J. M. Eibes G. (2017). Rutin: a review on extraction, identification and purification methods, biological activities and approaches to enhance its bioavailability. *Trends Food Sci. Technol.* 67 220–235. 10.1016/j.tifs.2017.07.008

[B15] Hagimori M. Kamiya S. Yamaguchi Y. Arakawa M. (2009). Improving frequency of thrombosis by altering blood flow in the carrageenan-induced rat tail thrombosis model. *Pharmacol. Res.* 60 320–323. 10.1016/j.phrs.2009.04.010 19394423

[B16] Hirsh J. Anand S. S. Halperin J. L. Fuster V. (2001a). Guide to anticoagulant therapy: heparin : a statement for healthcare professionals from the American Heart Association. *Circulation* 103 2994–3018. 10.1161/01.cir.103.24.2994 11413093

[B17] Hirsh J. Anand S. S. Halperin J. L. Fuster V. (2001b). Mechanism of action and pharmacology of unfractionated heparin. *Arterioscler. Thromb. Vasc. Biol.* 21 1094–1096. 10.1161/hq0701.093686 11451734

[B18] Ilinskaya A. N. Dobrovolskaia M. A. (2013a). Nanoparticles and the blood coagulation system. Part I: benefits of nanotechnology. *Nanomedicine (Lond.)* 8 773–784. 10.2217/nnm.13.48 23656264

[B19] Ilinskaya A. N. Dobrovolskaia M. A. (2013b). Nanoparticles and the blood coagulation system. Part II: safety concerns. *Nanomedicine (Lond.)* 8 969–981. 10.2217/nnm.13.49 23730696 PMC3939602

[B20] International Organization for Standardization (2009). *ISO 10993-5:2009. Biological evaluation of medical devices - Part 5: Tests for in vitro cytotoxicity.* Geneva: International Organization for Standardization.

[B21] International Organization for Standardization (2017). *ISO 10993-4:2017. Biological evaluation of medical devices - Part 4: Selection of tests for interactions with blood.* Geneva: International Organization for Standardization.

[B22] Jasuja R. Passam F. H. Kennedy D. R. Kim S. H. van Hessem L. Lin L. (2011). Protein disulfide isomerase inhibitors: a new class of antithrombotic agents. *Blood* 118:172 10.1182/blood.V118.21.369.369 PMC3366406 22565308

[B23] Jasuja R. Passam F. H. Kennedy D. R. Kim S. H. van Hessem L. Lin L. (2012). Protein disulfide isomerase inhibitors constitute a new class of antithrombotic agents. *J. Clin. Invest.* 122 2104–2113. 10.1172/jci61228 22565308 PMC3366406

[B24] Kargl R. Kleinschek K. S. (2020). How can we understand the influence of nanoparticles on the coagulation of blood? *Nanomedicine (Lond.)* 15 1923–1926. 10.2217/nnm-2020-0177 32677508

[B25] Kennedy D. R. Nag P. P. Galinski C. N. Bowley S. Bekendam R. H. Dilks J. R. (2013). Development of second generation thiol isomerase inhibitors to prevent thrombus formation. *Blood* 122:926 10.1182/blood.V122.21.926.926

[B26] Kyrle P. A. Minar E. Bialonczyk C. Hirschl M. Weltermann A. Eichinger S. (2004). The risk of recurrent venous thromboembolism in men and women. *N. Engl. J. Med.* 350 2558–2563. 10.1056/NEJMoa032959 15201412

[B27] Lin L. Gopal S. Sharda A. Passam F. Bowley S. R. Stopa J. (2015). Quercetin-3-rutinoside inhibits protein disulfide isomerase by binding to its b’x domain. *J. Biol. Chem.* 290 23543–23552. 10.1074/jbc.M115.666180 26240139 PMC4583019

[B28] Lippi G. Franchini M. Targher G. (2011). Arterial thrombus formation in cardiovascular disease. *Nat. Rev. Cardiol.* 8 502–512. 10.1038/nrcardio.2011.91 21727917

[B29] Mackman N. (2008). Triggers, targets and treatments for thrombosis. *Nature* 451 914–918. 10.1038/nature06797 18288180 PMC2848509

[B30] Piazza G. Goldhaber S. Z. Kroll A. Goldberg R. J. Emery C. Spencer F. A. (2012). Venous thromboembolism in patients with diabetes mellitus. *Am. J. Med.* 125 709–716. 10.1016/j.amjmed.2011.12.004 22560173 PMC3424058

[B31] Roy D. Sumerlin B. S. (2012). Glucose-sensitivity of boronic acid block copolymers at physiological pH. *ACS Macro Lett.* 1 529–532. 10.1021/mz300047c 35607054

[B32] Sharma S. Ali A. Ali J. Sahni J. K. Baboota S. (2013). Rutin: therapeutic potential and recent advances in drug delivery. *Expert Opin. Investig. Drugs* 22 1063–1079. 10.1517/13543784.2013.805744 23795677

[B33] Su M. Dai Q. Chen C. Zeng Y. Chu C. Liu G. (2020). Nano-medicine for thrombosis: a precise diagnosis and treatment strategy. *Nanomicro Lett.* 12:96 10.1007/s40820-020-00434-0 PMC7770919 34138079

[B34] Tejamaya M. Roemer I. Merrifield R. C. Lead J. R. (2012). Stability of citrate, pvp, and peg coated silver nanoparticles in ecotoxicology media. *Environ. Sci. Technol.* 46 7011–7017. 10.1021/es2038596 22432856

[B35] Thompson C. M. Occhialini G. McCandless G. T. Alahakoon S. B. Cameron V. Nielsen S. O. (2017). Computational and experimental studies on the effects of monomer planarity on covalent organic framework formation. *J. Am. Chem. Soc.* 139 10506–10513. 10.1021/jacs.7b05555 28696109

[B36] Tian Y. Zhao Y. Zheng W. Zhang W. Jiang X. (2014). Antithrombotic functions of small molecule-capped gold nanoparticles. *Nanoscale* 6 8543–8550. 10.1039/c4nr01937g 24965704

[B37] Wang Y. Zhou C. Sun L. Yu B. Cao M. Zhong S. (2015). One-step synthesis of boronic acid group modified silica particles by the aid of epoxy silanes. *Appl. Surf. Sci.* 351 353–357. 10.1016/j.apsusc.2015.05.120

[B38] Zahoor M. Shafiq S. Ullah H. Sadiq A. Ullah F. (2018). Isolation of quercetin and mandelic acid from *Aesculus indica* fruit and their biological activities. *BMC Biochem.* 19:5. 10.1186/s12858-018-0095-7 29940844 PMC6019818

[B39] Zhang Z. Zhang X. Xin Z. Deng M. Wen Y. Song Y. (2011). Synthesis of monodisperse silver nanoparticles for ink-jet printed flexible electronics. *Nanotechnology* 22:425601 10.1088/0957-4484/22/42/425601 21937786

[B40] Zhao L. Huang Q. Liu Y. Wang Q. Wang L. Xiao S. (2017). Boronic acid as glucose-sensitive agent regulates drug delivery for diabetes treatment. *Materials (Basel)* 10:170. 10.3390/ma10020170 28772528 PMC5459139

